# Temperature-Dependent Oviposition Models for *Monochamus saltuarius* (Coleoptera: Cerambycidae)

**DOI:** 10.3390/insects15080597

**Published:** 2024-08-06

**Authors:** Hyoseok Lee, Jong-Kook Jung, Youngwoo Nam, Sang-Hyun Koh

**Affiliations:** Division of Forest Insect Pests and Diseases, National Institute of Forest Science, Seoul 02455, Republic of Korea; hyoseok.lee@usda.gov (H.L.); shkoh@korea.kr (S.-H.K.)

**Keywords:** two-phase oviposition model, pine wood nematode, *Monochamus saltuarius*, pine wilt disease

## Abstract

**Simple Summary:**

Longhorn beetles of the genus *Monochamus* are vectors of the pine wood nematode, which causes incurable pine wilt disease in coniferous trees. We developed temperature-dependent models to describe the oviposition of *Monochamus saltuarius*, a significant species within this genus. Our study demonstrated that these models effectively capture the nonlinear effects of temperature on the oviposition and aging of *M. saltuarius* adults. We compared two simulation approaches: one that separately accounted for the sexual maturation phase of adults and another that did not. Both methods proved capable of accurately describing oviposition patterns across various temperature conditions. The insights gained from this study have potential applications beyond estimating oviposition and predicting population dynamics of *M. saltuarius*. This modeling framework could be adapted to forecast population changes in a diverse range of pest species, offering a versatile tool for pest management strategies.

**Abstract:**

*Monochamus saltuarius* Gebler is a serious insect pest in Europe and East Asia regions, including Portugal, Spain, China, Japan, and Korea. It transfers the pine wood nematode *Bursaphelenchus xylophilus* to conifer trees, resulting in pine wilt disease (PWD). As temperature is a key factor influencing insect population dynamics, temperature-dependent models describing *M. saltuarius* oviposition could estimate population growth potential and evaluate outbreak risks. In this study, the longevity and fecundity of *M. saltuarius* females were measured under constant temperature conditions ranging from 20 to 32 °C, and temperature-dependent models were constructed. The longevity of *M. saltuarius* females ranged from 83.36 days to 22.92 days, with a total fecundity of 141 eggs and 52.77 eggs at 20 °C and 32 °C, respectively. To describe oviposition, we used a single-phase simulation describing oviposition as a single model and a two-phase simulation describing sexual maturation and oviposition as two separate models. These models effectively described *M. saltuarius* oviposition (*r*^2^ > 0.96) under constant temperature conditions, with the two-phase simulation demonstrating greater accuracy overall. Such models could facilitate assessments of PWD risks. The modeling framework of this study shows potential for predicting threats from various forestry and agricultural pests.

## 1. Introduction

Pine wilt disease (PWD) is an important disease worldwide caused by the pine wood nematode *Bursaphelenchus xylophilus*. Coniferous species of *Abies*, *Cedrus*, *Larix*, *Picea*, *Pinus*, and *Pseudotsuga* genera are known to be hosts of *B. xylophilus* [1]. Native to North America, PWD has spread to Europe (e.g., Portugal and Spain) and East Asia (e.g., China, Japan, and Korea), causing fatal damage to various pine trees [2,3]. Over 13 species are known to transmit PWD, with longhorn beetles of the genus *Monochamus* being the most important vectors for *B. xylophilus* [4]. Seven *Monochamus* species in North America and three in Asia have been identified as potential PWD vectors, of which *M. saltuarius* and *M. alternatus* are the main vectors in China, Japan, and Korea [5,6]. Compared to *M. alternatus*, *M. saltuarius* is relatively more cold-adapted, inhabiting higher latitude regions [5]. Notably, PWD was recently first reported in Liaoning Province, northeast China, likely spread by *M. saltuarius* [4]. With climate change, cold-tolerant *M. saltuarius* appears to be expanding northward and increasing the risk of spreading PWD to new areas.

As with other ectotherms, temperature is likely the dominant environmental factor impacting insect biology, including key reproductive traits such as survival and fecundity [7]. Insect body temperature is mainly dependent on ambient temperature. Thus, climate change causing unprecedented thermal fluctuations and extreme events can significantly influence the population density of insects. Along with range expansion, outbreaks of insects are becoming more unpredictable with climate change [8]. Understanding *M. saltuarius* density changes is especially important for PWD management, as the density of this insect vector affects PWD epidemics [9].

Temperature influences insect survival and fecundity species-specifically. This has been described by various models based on experiments [10]. In particular, multiple temperature-dependent models, including adult survival, total fecundity, and oviposition rate models, have been combined to estimate daily oviposition under varying temperatures. Although oviposition stage could be divided into sexual maturation (pre-oviposition) and oviposition phases, both phases are temperature-sensitive and often described by a unified model (single-phase simulation) [11,12]. However, the shape of a unified model may erroneously depict oviposition occurring during sexual maturation phase. To address this, using a two-phase simulation by separating sexual maturation and oviposition could enable more realistic fecundity estimates [13]. Such a simulation can estimate potential population growth across generations and quantitatively assess outbreak risk.

*M. saltuarius* overwinters as mature larvae and emerges as adults in the following spring [14]. After emergence, they undergo a sexual maturation period of 1–2 weeks before oviposition [15]. Female adults excavate slits in the bark of pine tree boughs and trunks to lay their eggs. *M. saltuarius* adults that emerge from infected trees transmit *B. xylophilus* to healthy pine trees during their feeding and oviposition processes [16]. This study investigated the lifespan and fecundity of newly emerged *M. saltuarius* female adults reared under constant temperature conditions and developed temperature-dependent oviposition models. Models for single-phase and two-phase simulations were constructed, and their performances were compared. These results will aid in our understanding of the relationship between temperature and oviposition in *M. saltuarius*. They could be used to predict population density changes to enhance PWD management.

## 2. Materials and Methods

### 2.1. Oviposition Experiments

*M. saltuarius*-infested Korean white pine trees (*Pinus koraiensis*) were logged from pine stands in Hongcheon-gun, Gangwon-do, Korea (37.76102° in latitude, 127.8895° in longitude), in December 2014. These logged trees were kept in a semi-field emergence cage in the National Institute of Forest Science, Seoul, Korea. Newly emerged adults (<24 h) were collected daily and used for oviposition experiments. Individual pairs of newly emerged adults were transferred into cylindrical plastic containers (15 cm in diameter, height of 20 cm). Korean white pine tree trunks were cut into pine bolts. Cut ends were coated with paraffin to prevent desiccation [15]. One coated pine bolt, additional twigs, and a watered cotton ball were provided to each pair of *M. saltuarius*. These containers were kept in environmental chambers (DS-8CL, Dasol Scientific; Hwaseong, Republic of Korea) set at five constant temperatures (20, 24, 28, 30, and 32 °C) with a photoperiod of 14:10 h (light/dark). Survival of female adults was monitored every day. Male adults that died during early stages of the experiment (<1 week) were replaced with new male adults to ensure mating. Coated pine bolts were replaced every three days. After removing all the bark from the collected pine bolts, we counted the number of eggs under a binocular stereomicroscope (Stemi 305, Carl Zeiss, Jena, Germany) until females died. Females that died within 7 days were not included in the analysis.

Total fecundity, oviposition rate, female longevity, and pre-oviposition period (i.e., period from emergence to first oviposition) were measured. An analysis of variance was conducted to determine differences. Normality of all datasets was verified using the Shapiro–Wilk test. Mean values were compared through Tukey’s honest significance test. All statistical analyses were conducted using R 4.3.1 [17].

### 2.2. Model Development

We developed two forms of simulation processes to describe oviposition of *M. saltuarius*. The single-phase simulation consisted of one model describing oviposition rate from adult emergence. The two-phase simulation was comprised of two separate models, one depicting sexual maturation after adult emergence (pre-oviposition) and the other describing oviposition thereafter (oviposition). Physiological age of adults was estimated using a temperature-dependent aging model. In addition, lifetime total fecundity of female adults was described through a temperature-dependent total fecundity model. In the single-phase simulation, daily oviposition was estimated based on models of age-dependent survival rate and cumulative oviposition rate. In the two-phase simulation, the same temperature-dependent aging, total fecundity, and age-dependent survival rate models were employed. However, daily oviposition was determined by first estimating the period to sexual maturity using a temperature-dependent development model, and then by applying a cumulative oviposition rate model.

### 2.3. Temperature-Dependent Aging Model and Age-Dependent Model

Aging rate (1/mean longevity) of female adults is temperature-dependent. It demonstrates a nonlinear relationship with temperature [18]. In contrast to nymphs that show a sharp decrease in development rate beyond optimum temperature, the aging rate of adults increases exponentially as temperature rises. Hence, we used the following exponential function to describe the temperature-dependent aging rate of female adults:

(1) $$A\left(T\right)=r_{0}+\exp[\frac{T-T_{H}}{c}]$$

where *A*(*T*) is the aging rate (1/day) at temperature *T* (°C), *r*_0_ is the minimum aging rate, and *T_H_* and *c* are estimated parameters. As longevity varied across temperatures, we normalized daily aging rates using the aging rate model, with cumulative daily values of aging rates giving physiological ages of adults. That is, the physiological age reaches 1 at the mean longevity of adults at each temperature [19].

The proportion of surviving females to total number of female adults was used to describe survival of female adults. In addition, survival rate was normalized based on the physiological age for comparison across temperature conditions. Age-specific survival rate (*S*(*P_x_*)) at a physiological age (*P_x_*) was described using a two-parameter Weibull function:

(2) $$S\left(P_{x}\right)=exp[-{\left(P_{x}/\alpha \right)}^{\beta }]$$

where *α* and *β* are estimated parameters, with *α* denoting the physiological age of 50% of the surviving adults and *β* determining the shape of the curve.

### 2.4. Temperature-Dependent Fecundity Model and Cumulative Oviposition Model

A temperature-dependent total fecundity model was developed to describe the relationship between lifetime fecundity and temperature. Total fecundity over temperature resembled a bell-shaped nonlinear relationship. It was fit by using the Briere-1 function:

(3) $$F\left(T\right)=\alpha \cdot (T-T_{L})\cdot (T_{H}-T)$$

where *F*(*T*) is the total number of eggs yielded over the entire lifespan by a female at temperature *T* (°C) with low (*T_L_*) and high (*T_H_*) temperature limits, and *α* is a fitted constant. For model development, we assumed 10.1 °C as the low threshold temperature for oviposition [20], added this data point to our dataset, and then estimated *α*, *T_L_*, and *T_H_* parameters by model fitting.

The cumulative proportion of eggs laid was modeled to characterize the oviposition rate of *M. saltuarius*. The cumulative oviposition model was normalized by physiological age to allow for a comparison across different physiological ages for multiple temperature conditions. The cumulative oviposition rate was described using a two-parameter Weibull function:

(4) $$E\left(P_{x}\right)=1-\exp[-{\left(\frac{P_{x}}{\eta }\right)}^{\beta }]$$

where *E*(*P_x_*) is the cumulative proportion of eggs laid by a female at physiological age (*P_x_*), and *η* and *β* are the estimated parameters. Daily oviposition rate was defined as the incremental change in cumulative oviposition rate over time. For example, oviposition rate at day *t* was *E*(*P_xt_*) − *E*(*P_xt_*_−1_).

### 2.5. Sexual Development Rate and Completion Distribution Models

Sexual development rate and completion distribution models were developed for a two-phase simulation. Temperature-dependent sexual development rate was characterized using the Briere-2 model:

(5) $$D_{pre}\left(T\right)=\alpha \cdot T\cdot (T-T_{L})\cdot {\left(T_{H}-T\right)}^{\frac{1}{2}}$$

where *D_pre_*(*T*) is the development rate of sexually immature female adults, and *T_L_* and *T_H_* are the lower and upper temperature limits, respectively. For model fitting, *T_H_* was predetermined to be 38.79 °C [21]. Parameters *α* and *T_L_* are to be fitted. Physiological age for sexually immature females was accumulated using the temperature-dependent sexual development rate model. To compare across physiological ages, duration distribution to sexual maturation was described using a two-parameter Weibull function (Equation (4)). In the simulation, no oviposition was presumed before completion of sexual maturation.

The aging rate for oviposition upon sexual maturity was expressed as the reciprocal of the total oviposition period and described by an exponential function (Equation (1)). Aging rate for oviposition was accumulated to measure the physiological age for oviposition. Age-specific cumulative oviposition probability distribution was described by a two-parameter Weibull function (Equation (2)). To assess potential temperature-dependent differences in the cumulative oviposition probability, we employed a mixed-effects model using the ‘lme4’ package. The model was constructed with age and temperature as fixed effects, including their interaction, and a random intercept for each experimental unit.

Both single-phase and two-phase simulations were performed to estimate daily oviposition of *M. saltuarius* under multiple temperature conditions. Model parameters were estimated using ‘nls’ and ‘nls2’ functions. All simulations were performed in R 4.3.1 [17].

## 3. Results

**Figure 1:**
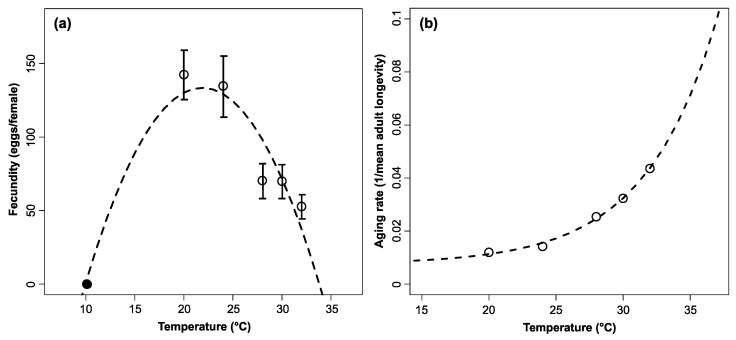
Temperature-dependent total fecundity (eggs/female) (**a**) and aging rate (1/mean days of longevity) (**b**) models for *Monochamus saltuarius* female adults. Exponential and Briere-1 functions were fitted to aging rate and total fecundity, respectively.

Temperature significantly influenced the fecundity and longevity of *M. saltuarius* females (Table 1). Eggs were laid at all experimental temperatures. Among the temperatures tested in this study, the fecundity of *M. saltuarius* peaked at 20 °C, with a total of 141 eggs laid per female. Fecundity decreased with increasing temperature over the tested range, and this relationship was well described by the Briere-1 model, which assumed no oviposition below 15 °C [22] (*F*_2,3_ = 5.60, *p* < 0.001, *R*^2^ = 0.789) (Figure 1a and Table 2). In contrast, female longevity decreased exponentially with increasing temperature (Table 1). *M. saltuarius* females showed an average longevity of 83.36 days at 20 °C and 22.92 days at 32 °C. The temperature-dependent aging rate of *M. saltuarius* females was estimated as the inverse of mean longevity. Aging rate increased exponentially with temperature (*F*_2,2_ = 228.51, *p* = 0.004, *R*^2^ = 0.996) (Figure 1b and Table 2).

**Figure 2:**
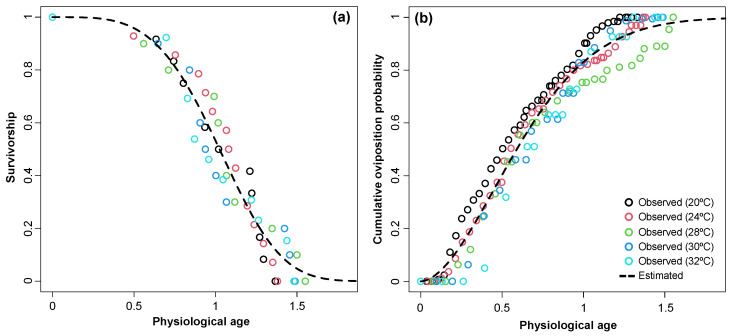
Physiological age-specific survivorship (**a**) and cumulative oviposition probability curves (**b**). A two-parameter Weibull function was used to estimate physiological age-specific survivorship and cumulative oviposition probability.

Physiological age, the accumulation of aging rate, was used to compare female age-specific survival and cumulative oviposition. There were no significant differences in the patterns of cumulative survival change over age among the temperatures (survival: *X*^2^ = 0.78, *df* = 4; *p* = 0.94; oviposition: *X*^2^ = 2.45, *df* = 4; *p* = 0.12). Physiological age-dependent survival was well described by the Weibull function (*F*_1,53_ = 869.24, *p* < 0.0001, *R*^2^ = 0.943) (Figure 2a and Table 2), with 53% survival at mean longevity (i.e., physiological age = 1). The relationship between cumulative oviposition and physiological age was well described by the two-parameter Weibull function (*F*_1,153_ = 4414.7, *p* < 0.0001, *R*^2^ = 0.967) (Table 2), with 83.2% of lifetime fecundity predicted to occur by mean longevity.

**Figure 3:**
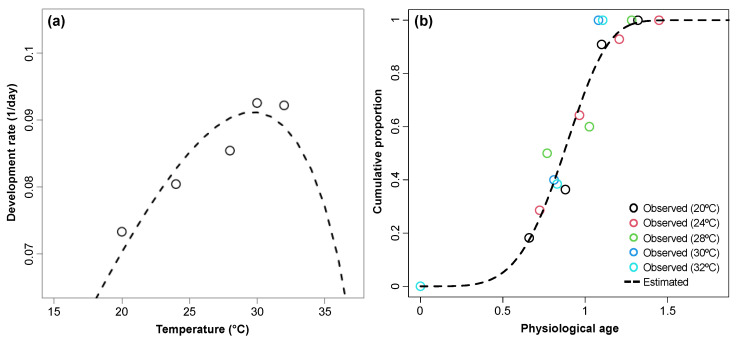
Temperature-dependent development rate for sexual maturation (**a**) and cumulative proportion of age-specific development completion (**b**) for sexual maturation of *Monochamus saltuarius* female adults. Briere-2 and two-parameter Weibull functions were applied to estimate development rate and cumulative proportion, respectively.

The developmental rate for sexual maturation was estimated as the inverse of the pre-oviposition period. The relationship between temperature and sexual maturation rate was described by the Briere-2 model (*F*_1,3_ = 12.936, *p* = 0.037, *R*^2^ = 0.812) (Figure 3a and Table 2). Using this relationship, physiological age for sexual maturation was separately estimated. The relationship between physiological age and cumulative proportion of sexual maturation was described by the Weibull function (*F*_1,18_ = 411.71, *p* < 0.0001, *R*^2^ = 0.960) (Figure 3b and Table 2).

**Figure 4:**
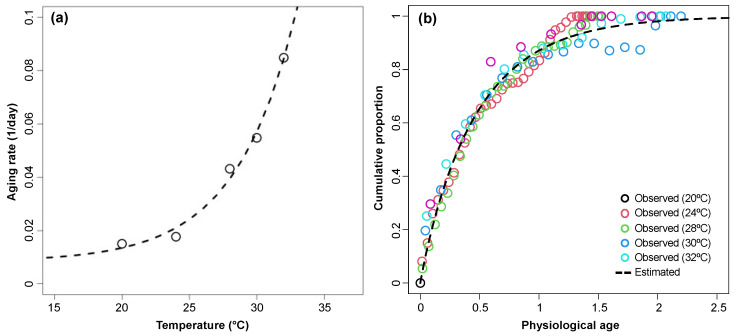
Temperature-dependent development rate for oviposition (**a**) and age-specific cumulative oviposition probability (**b**) of sexually matured *Monochamus saltuarius* female adults. An exponential function and a two-parameter Weibull function were applied to estimate development rate and cumulative oviposition probability, respectively.

Following the sexual maturation phase, aging rate during the oviposition phase was estimated as the inverse of the period from first oviposition until female death. The relationship between temperature and aging rate was described by an exponential function (*F*_2,2_ = 98.493, *p* = 0.010, *R*^2^ = 0.990) (Figure 4a and Table 2). Cumulative oviposition rate was estimated by physiological age following sexual maturation. The relationship between cumulative oviposition rate and physiological age was well described by the Weibull function (*F*_1,111_ = 3724.9, *p* < 0.0001, *R*^2^ = 0.971) (Figure 4b and Table 2).

**Figure 5:**
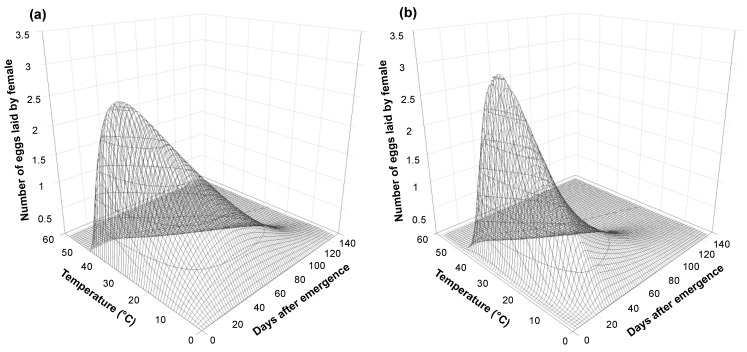
Predicted daily egg production of *Monochamus saltuarius* in relation to days after emergence and temperature by single-phase (**a**) and two-phase (**b**) simulations.

**Figure 6:**
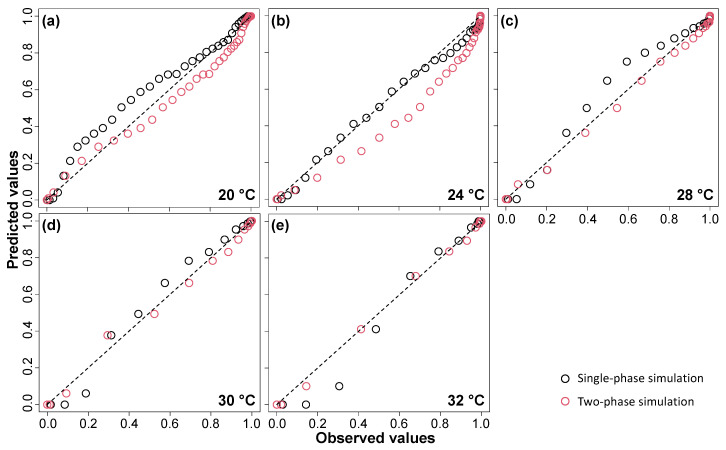
Comparison of simulation outputs from single-phase and two-phase simulations with the observed cumulative proportion of oviposition at various constant temperatures. Subfigures (**a**–**e**) represent different constant temperature conditions: 20, 24, 28, 30, and 32 °C, respectively.

Estimated models were used to predict daily oviposition from 10 to 40 °C by single- and two-phase simulations (Figure 5). The two-phase simulation predicted a shorter period of intensive oviposition compared to the single-phase simulation. Peak daily oviposition was predicted at 37 and 33 °C in the single-phase and two-phase simulations, respectively. Outputs of single-phase and two-phase simulations were compared to the observed cumulative proportions of oviposition at each temperature condition (Figure 6). Both simulations described daily oviposition well across all temperature ranges (Table 3). At all temperatures except 24 °C, the two-phase simulation demonstrated higher predictive power than the single-phase simulation.

## 4. Discussion

Female *M. saltuarius* adults were affected by temperature in terms of longevity, sexual maturation, aging rate, and oviposition. Based on the relationships between these traits and temperature, we were able to develop temperature-dependent models and describe the oviposition of *M. saltuarius* through simulations that connected these models. In this study, five constant temperature conditions (20, 24, 28, 30, and 32 °C) were used to develop temperature-dependent models. However, for total fecundity, there were limitations in covering the low-temperature range where nonlinear correlations with temperature were observed. Nevertheless, we were able to describe these nonlinear relationships with temperature through existing literature data [20]. Insect fecundity is an important factor that can be used to predict potential increases in population density. Temperature is one of the most influential factors affecting insect fecundity. Numerous studies have examined temperature–fecundity relationships under different temperature regimes. Here, we showed that under laboratory conditions, the relationship between fecundity and temperature followed a unimodal shape, increasing up to an optimum and then decreasing. Here, we demonstrated that, when combined with existing data [20], the relationship between fecundity and temperature followed a unimodal shape under laboratory conditions, increasing up to an optimum and then decreasing. However, the longevity of *M. saltuarius* female adults decreased with increasing temperature. We also fit nonlinear models describing these relationships that could enable simulating the daily oviposition of *M. saltuarius* by two types of simulations (i.e., single-phase and two-phase) across diverse temperature conditions.

Newly emerged *M. saltuarius* adults require a pre-oviposition development period before mating for oviposition [15]. Temperature-dependent oviposition models can incorporate this period in different ways. Some models combine pre-oviposition and oviposition phases into a single oviposition model [11,12], while others treat them as distinct phases [13]. In this study, we compared single-phase and two-phase simulations for describing the oviposition of *M. saltuarius*. Our results suggested that both models fit the oviposition similarly well for *M. saltuarius*. This may be due to the relatively short pre-oviposition period observed in this species (13.64 days at 20 °C and 10.85 days at 32 °C). However, the relative performance of single-phase versus two-phase simulations may vary depending on the duration of the pre-oviposition period in different species. For instance, in species with longer pre-oviposition periods, the differences between these two approaches might be more pronounced. For example, the pre-oviposition period of the oriental fruit fly was 38.1 and 6.2 days at 16.7 and 34.9 °C, respectively, showing significant differences compared to single-phase simulation outputs [13]. The choice between single-phase and two-phase simulations may depend on the pre-oviposition period of the species being studied and the level of detail required in the simulation. Further comparative studies across different insect species could provide more insights into the relative strengths and limitations of these modeling approaches.

As a native species in Korea, *M. saltuarius* emerges from May to June [14], with oviposition occurring mainly from June to August. Later-emerging individuals may experience higher temperatures, potentially resulting in lower fecundity. However, variations in life history traits among local populations may lead to differences in emergence timing [21]. For more accurate predictions of specific populations in narrow areas, model calibration may be required. The nonlinear models developed in this study can account for seasonal temperature changes. However, to incorporate daily temperature fluctuations, the current models’ time scale could be converted from daily to hourly.

Various techniques have been used to describe insect oviposition, including life table parameters and degree day models [23,24]. In this study, both single- and two-phase simulations predicted daily oviposition close to experimental observations under the constant temperature conditions (*R*^2^ > 0.96). Temperature-dependent models offer the advantage of the experimental determination of responses to temperature under different constant temperature regimes, enabling prediction across fluctuating temperatures through nonlinear regression and simulation [18]. Simulating such models under thermal variation relies on the assumption that individuals exhibit identical responses at a given physiological age and temperature irrespective of the previously experienced thermal path. However, temperature responses are likely to differ with given developmental and nutritional status as well as physiological age [25,26]. Physiological age was used to standardize relative age (days) and incorporate temperature responses into nonlinear models, although it may also be influenced by other factors (e.g., diet, abiotic/biotic stress) and the environment experienced by individuals [27]. Furthermore, the ambient temperature conditions may vary depending on the development rate and the timing of diapause termination, which can consequently affect fecundity. Accordingly, diverse environmental validation is essential to evaluate model robustness.

Various model-based forecasting approaches have facilitated our understanding of target pests and enabled practical pest management applications. Phenology models could be used to optimize control timing for target pests, while fecundity models may be used to assess seasonal outbreak potential. Additionally, combining our fecundity models with phenology and winter survival models might enable predictions of population dynamics of *M. saltuarius*. Furthermore, integrating these models with existing species distribution models (SDMs) [28] could contribute to the development of a comprehensive risk index that can estimate both the distribution and abundance of *M. saltuarius* [29]. Given the importance of *M. saltuarius* as a PWD vector in East Asia, such models could be used to forecast the timing of disease transmission by adults and provide enhanced resolution of density changes to evaluate epidemic risk. This framework can be expanded to more precisely predict threats and dynamics of various forest and agricultural pests.

## Figures and Tables

**Table 1:** Fecundity and longevity (days) of *Monochamus saltuarius* female adults at various constant temperatures.

Temperature (°C)	n	Longevity (Days)	Pre-oviposition Period (Days)	Fecundity (Eggs/Female)
20	12	83.36 ± 5.31 ^a^	13.64 ± 0.85	141 ± 18.41 ^a^
24	14	70.29 ± 4.89 ^a^	12.43 ± 0.76	134.71 ± 20.84 ^a^
28	10	39.3 ± 4.27 ^b^	11.70 ± 0.94	83.2 ± 13.87 ^ab^
30	10	30.9 ± 2.86 ^bc^	10.80 ± 0.49	70 ± 11.52 ^b^
32	13	22.92 ± 1.23 ^c^	10.85 ± 0.42	52.77 ± 8.09 ^b^

Means followed by the same letter within a column are not significantly different (*p* > 0.05, Tukey’s studentized range test).

**Table 2:** Parameter estimates (±S.E.) of models for single-phase and two-phase simulations.

Models		Parameter	Estimate ± S.E.	*p*	*R* ^2^
Single-phase	Aging rate	*r*_0_	0.0075 ± 0.0025	0.0966	0.997
		*T_H_*	49.6674 ± 2.7553	0.0031	
		*c*	5.3255 ± 0.9031	0.0276	
	Fecundity	*α*	0.937 ± 0.1712	0.0120	0.915
		*T_L_*	9.944 ± 0.9087	0.0016	
		*T_H_*	33.812 ± 0.9863	<0.0001	
	Survival	*α*	1.1269 ± 0.0119	<0.0001	0.944
		*β*	3.8881 ± 0.2440	<0.0001	
	Cumulative oviposition	*η*	0.7246 ± 0.0078	<0.0001	0.967
		*β*	1.7824 ± 0.0539	<0.0001	
Two-phase	Development rate for sexual maturation	*α*	2.147 × 10^−5^ ± 4.902 × 10^−6^	0.022	0.849
		*T_L_*	−17.74 ± 10.20	0.180	
	Age-specific development completion for sexual maturation	*η*	0.9418 ± 0.0178	<0.0001	0.960
		*β*	4.6427 ± 0.5871	<0.0001	
	Aging rate for oviposition	*r*_0_	0.0084 ± 0.0064	0.3184	0.992
		*T_H_*	43.4441 ± 2.4164	0.0031	
		*c*	4.4394 ± 1.0140	0.0484	
	Age-specific cumulative oviposition rate	*η*	0.4693 ± 0.0088	<0.0001	0.971
		*β*	0.9482 ± 0.0263	<0.0001	

**Table 3:** Comparison of adjusted *R*^2^ values between single-phase and two-phase simulation outputs at various constant temperatures (***, highly significant with *p* < 0.001).

Temperature (°C)	Adjusted *R*^2^	
	Single-Phase	Two-Phase
20	0.980 ***	0.985 ***
24	0.995 ***	0.963 ***
28	0.974 ***	0.998 ***
30	0.984 ***	0.995 ***
32	0.981 ***	0.999 ***
Average	0.983	0.988

## Data Availability

The data are available upon request.

## References

[1] Schenk M., Loomans A., den Nijs L., Hoppe B., Kinkar M., Vos S., European Food Safety Authority (2020). Pest Survey Card on *Bursaphelenchus xylophilus*. EFSA Support. Publ..

[2] Mamiya Y. (1983). Pathology of the Pine Wilt Disease Caused by *Bursaphelenchus xylophilus*. Annu. Rev. Phytopathol..

[3] Vicente C., Espada M., Vieira P., Mota M. (2012). Pine Wilt Disease: A Threat to European Forestry. Eur. J. Plant Pathol..

[4] Li M., Dai Y., Wang Y., Wang L., Sun S., Chen F. (2021). New Insights into the Life History of *Monochamus saltuarius* (Cerambycidae: Coleoptera) Can Enhance Surveillance Strategies for Pine Wilt Disease. J. For. Res..

[5] Kwon T.-S., Lim J.-H., Sim S.-J., Kwon Y.-D., Son S.-K., Lee K.-Y., Kim Y.-T., Park J.-W., Shin C.-H., Ryu S.-B. (2006). Distribution Patterns of *Monochamus alternatus* and *M. saltuarius* (Coleoptera: Cerambycidae) in Korea. J. Korean For. Soc..

[6] Xu Q., Zhang X., Li J., Ren J., Ren L., Luo Y. (2023). Pine Wilt Disease in Northeast and Northwest China: A Comprehensive Risk Review. Forests.

[7] Bale J.S., Masters G.J., Hodkinson I.D., Awmack C., Bezemer T.M., Brown V.K., Butterfield J., Buse A., Coulson J.C., Farrar J. (2002). Herbivory in Global Climate Change Research: Direct Effects of Rising Temperature on Insect Herbivores. Glob. Chang. Biol..

[8] Harvey J.A., Heinen R., Gols R., Thakur M.P. (2020). Climate Change-Mediated Temperature Extremes and Insects: From Outbreaks to Breakdowns. Glob. Chang. Biol..

[9] Kwon T.-S., Shin J.H., Lim J.-H., Kim Y.-K., Lee E.J. (2011). Management of Pine Wilt Disease in Korea through Preventative Silvicultural Control. For. Ecol. Manag..

[10] Régnière J., Powell J., Bentz B., Nealis V. (2012). Effects of Temperature on Development, Survival and Reproduction of Insects: Experimental Design, Data Analysis and Modeling. J. Insect Physiol..

[11] Baek S., Hwang A., Kim H., Lee H., Lee J.-H. (2017). Temperature-Dependent Development and Oviposition Models of *Halyomorpha halys* (Hemiptera: Pentatomidae). J. Asia. Pac. Entomol..

[12] Sampaio F., Batista M.M., Marchioro C.A. (2024). Temperature-Dependent Reproduction of *Spodoptera eridania*: Developing an Oviposition Model for a Novel Invasive Species. Pest Manag. Sci..

[13] Choi K.S., Samayoa A.C., Hwang S.-Y., Huang Y.-B., Ahn J.J. (2020). Thermal Effect on the Fecundity and Longevity of *Bactrocera dorsalis* Adults and Their Improved Oviposition Model. PLoS ONE.

[14] Han J.-H., Yoon C., Shin S.-C., Kim G.-H. (2007). Seasonal Occurrence and Morphological Measurements of Pine Sawyer, *Monochamus saltuarius* Adults (Coleoptera: Cerambycidae). J. Asia. Pac. Entomol..

[15] Nakayama Y., Jikumaru S., Togashi K. (1998). Reproductive Traits and Diel Activity of Adult *Monochamus saltuarius* (Coleoptera: Cerambycidae) at Two Different Temperatures. J. For. Res..

[16] Jikumaru S., Togashi K. (2001). Transmission of *Bursaphelenchus Mucronatus* (Nematoda: Aphelenchoididae) through Feeding Wounds by *Monochamus saltuarius* (Coleoptera: Cerambycidae). Nematol..

[17] R core team (2023). R: A Language and Environment for Statistical Computing.

[18] Rebaudo F., Rabhi V.-B. (2018). Modeling Temperature-Dependent Development Rate and Phenology in Insects: Review of Major Developments, Challenges, and Future Directions. Entomol. Exp. Appl..

[19] Wagner T.L., Wu H.-I., Sharpe P.J.H., Coulson R.N. (1984). Modeling Distributions of Insect Development Time: A Literature Review and Application of the Weibull Function. Ann. Entomol. Soc..

[20] Jikumaru S., Togashi K. (1996). Effect of Temperature on the Post-Diapause Development of *Monochamus saltuarius* (GEBLER) (Coleoptera: Cerambycidae). Appl. Entomol. Zool..

[21] Jung C.S., Koh S.-H., Nam Y., Ahn J.J., Lee C.Y., Choi W.I. (2015). A Model for Predicting Spring Emergence of *Monochamus saltuarius* (Coleoptera: Cerambycidae) from Korean White Pine, *Pinus koraiensis*. J. Econ. Entomol..

[22] Yoon S., Jung J.-M., Hwang J., Park Y., Lee W.-H. (2023). Ensemble Evaluation of the Spatial Distribution of Pine Wilt Disease Mediated by Insect Vectors in South Korea. For. Ecol. Manag..

[23] Zanuncio J.C., Lemos W.P., Lacerda M.C., Zanuncio T.V., Serrão J.E., Bauce E. (2006). Age-Dependent Fecundity and Fertility Life Tables of the Predator *Brontocoris tabidus* (Heteroptera: Pentatomidae) under Field Conditions. J. Econ. Entomol..

[24] Blatt S., Joseph D.A., Cutler G.C., Olson A.R., White S. (2020). Degree-Day Models to Predict Carrot Weevil (Coleoptera: Curculionidae) Emergence and Oviposition in Nova Scotia, Canada. Can. Entomol..

[25] Nyamukondiwa C., Terblanche J.S. (2009). Thermal Tolerance in Adult Mediterranean and Natal Fruit Flies (*Ceratitis capitata* and *Ceratitis rosa*): Effects of Age, Gender and Feeding Status. J. Therm. Biol..

[26] Scaccini D., Duso C., Pozzebon A. (2019). Lethal Effects of High Temperatures on Brown Marmorated Stink Bug Adults before and after Overwintering. Insects.

[27] Boggs C.L. (2009). Understanding Insect Life Histories and Senescence through a Resource Allocation Lens. Funct. Ecol..

[28] Kim J., Jung H., Park Y.-H. (2016). Predicting Potential Distribution of *Monochamus alternatus* Hope Responding to Climate Change in Korea. Korean J. Appl. Entomol..

[29] Mi C., Huettmann F., Sun R., Guo Y. (2017). Combining Occurrence and Abundance Distribution Models for the Conservation of the Great Bustard. PeerJ.

